# The effect of childhood maltreatment on non-suicidal self-injury in adolescents with bipolar disorder: the mediating role of dysfunctional attitudes and the moderating role of social support

**DOI:** 10.3389/fpsyt.2025.1698424

**Published:** 2025-12-10

**Authors:** Huawei Tan, Dan Zhao, Jiarui Cao, Ting Huang, Jiahui Yi, Zhihui Wan, Fan Zhang

**Affiliations:** 1 Department of Psychiatry, Renmin Hospital of Wuhan University, Wuhan, Hubei, China; 2 Department of Rehabilitation, Renmin Hospital of Wuhan University, Wuhan, Hubei, China; 3 Department of Orthopedics, Renmin Hospital of Wuhan University, Wuhan, Hubei, China; 4 Department of Nursing, Renmin Hospital of Wuhan University, Wuhan, Hubei, China

**Keywords:** childhood maltreatment, nonsuicidal self-injury, bipolar disorder, dysfunctional attitudes, social support

## Abstract

**Background:**

Childhood maltreatment has been consistently associated with nonsuicidal self-injury (NSSI). Patients with bipolar disorder (BD) are especially vulnerable to early adversity and self-injurious behaviors; however, the mechanisms underlying this association remain unclear. Among Chinese patients with BD, this study tested whether dysfunctional attitudes mediate the association between childhood maltreatment and NSSI and whether social support moderates this association.

**Methods:**

A cross-sectional study was conducted with 838 clinically diagnosed bipolar disorder patients (68.1% female; median age = 16 years). Measures included the Childhood Trauma Questionnaire–Short Form (CTQ-SF), Dysfunctional Attitudes Scale (DAS-A), Social Support Rating Scale (SSRS), Ottawa Self-Injury Inventory–Chinese Revised Edition (OSIC), and Hamilton Depression Rating Scale (HAMD-24). Descriptive analyses, correlations, mediation, and moderated mediation models were tested using SPSS 26.0 and Mplus 8.3, with robust maximum likelihood estimation (MLR) and 5,000 bootstrap resamples.

**Results:**

In our clinical sample of patients with bipolar disorder, 68.5% reported nonsuicidal self-injury (NSSI). Childhood maltreatment was positively associated with both dysfunctional attitudes and NSSI. Mediation analysis demonstrated that dysfunctional attitudes partially mediated the association between childhood maltreatment and NSSI (indirect effect β = 0.040, 95% CI [0.017, 0.061], p < 0.01), accounting for 18.2% of the total effect. Moderation analysis further indicated that social support significantly attenuated the association between dysfunctional attitudes and NSSI (interaction β = –0.082, 95% CI [–0.162, –0.015], p < 0.05), but did not moderate the associations of childhood maltreatment with either dysfunctional attitudes or NSSI.

**Conclusion:**

Childhood maltreatment increases the risk of NSSI in patients with BD both directly and indirectly through dysfunctional attitudes, while social support mitigates the behavioral impact of dysfunctional attitudes on NSSI. These findings highlight dysfunctional attitudes as a cognitive mechanism and social support as a conditional protective factor, underscoring the importance of childhood maltreatment screening, cognitive restructuring, and support-enhancing interventions in reducing NSSI risk among Chinese patients with bipolar disorder.

## 1 Introduction

Bipolar disorder (BD) is a chronic and recurrent psychiatric condition recognized by alternating episodes of depression and mania or hypomania, with self-harm behavior and elevated suicide risk being among the most critical clinical challenges (1). Nonsuicidal self-injury (NSSI) refers to the deliberate and direct destruction of one’s own bodily tissue without suicidal intent, and it has emerged as a prevalent and clinically relevant behavior within psychiatric populations (2). NSSI is highly prevalent among individuals with BD, with lifetime rates ranging from 40% to 50% (3, 4). Recent data from China reports rates over 40% overall and up to 78% among adolescents, highlighting the urgent need to understand its risk factors (5). Beyond its high prevalence, NSSI in BD is associated with increased suicide attempts, greater illness severity, higher hospitalization rates, and impaired psychosocial functioning, underscoring its clinical and public health significance (4, 6). Therefore, identifying the potential mechanisms underlying NSSI in BD is crucial for informing effective prevention and intervention strategies.

Childhood maltreatment (CM), encompassing emotional, physical, and sexual abuse as well as neglect, is a pervasive adverse experience that profoundly disrupts psychological development and increases vulnerability to maladaptive behaviors (7). Beyond its high prevalence in the general population, CM is particularly common among individuals with BD, with meta-analytic evidence showing that around one-third report at least one form of abuse or neglect (8). Empirical studies have consistently demonstrated that adolescents exposed to CM are more likely to report frequent and severe NSSI compared with their non-maltreated peers (9, 10). Evidence from Chinese clinical samples indicates that CM is an important risk factor for NSSI among individuals with BD (11). Evidence further suggests that different forms of CM may exert differential effects, with emotional abuse and neglect particularly associated with elevated risk of self-injurious behaviors (12). Despite these findings, the mechanisms underlying the link between CM and NSSI remain insufficiently understood. Most studies have focused on direct associations, whereas the cognitive mechanisms linking CM to NSSI remain unclear. Clarifying these mechanisms may help identify cognitive risk factors and guide targeted interventions for adolescents with BD.

Dysfunctional attitudes, conceptualized as rigid, maladaptive cognitive schemas concerning self-worth, achievement, and approval, have been implicated in the onset and maintenance of depressive symptoms (13). According to the cognitive vulnerability model, early adverse experiences may foster the development of such maladaptive schemas, which increase susceptibility to psychopathology when activated by stress (14). In adult patients with BD, CM has been shown to exacerbate dysfunctional attitudes by fostering cognitive distortions such as hopelessness and overgeneralization, thereby strengthening cognitive vulnerability to depression (15). Existing evidence from major depressive disorder(MDD) samples indicates that adolescents with NSSI exhibit elevated dysfunctional attitudes, supporting dysfunctional attitudes as a cognitive risk factor for self-injurious behaviors (16). Taken together, these findings suggest that dysfunctional attitudes may serve as a potential mediator linking CM to NSSI. Although these studies provide important insights into how CM may contribute to cognitive vulnerability and NSSI, the evidence has come largely from MDD populations. Whether similar mechanisms operate in adolescents with BD remains unclear, particularly given the cognitive features unique to BD. Clarifying this pathway is essential for refining trauma–cognition–behavior models and for developing mechanism-based interventions tailored to high-risk clinical populations, particularly patients with BD. Therefore, the present study aimed to address this gap by examining the potential mediating role of dysfunctional attitudes in the association between CM and NSSI among individuals with BD.

Social support, encompassing perceived emotional, instrumental, and informational assistance from significant others, has been shown to play a critical protective role in individuals with BD (17). According to the stress-buffering model (18), social support protects individuals from the negative health consequences of stress by shaping cognitive appraisals, strengthening coping resources, and fostering adaptive rather than maladaptive responses. Consistent with this theoretical framework, both subjective and objective dimensions of social support have been identified as important protective resources in BD, facilitating remission, reducing relapse risk, and enhancing psychosocial functioning (19, 20). Empirical studies further demonstrate that higher levels of social support significantly weaken the association between CM and NSSI, suggesting a buffering effect whereby social support reduces the likelihood that early adversity translates into self-injurious behaviors (21, 22). Related findings also indicate that perceived social support can buffer the link between maladaptive cognitive emotion regulation and suicidal ideation, underscoring its broader protective role in maltreated populations (23).

However, whether social support moderates the cognitive pathway from CM to dysfunctional attitudes and the subsequent behavioral pathway from dysfunctional attitudes to NSSI remains unexplored, despite dysfunctional attitudes being a well-documented cognitive vulnerability; moreover, this question has not yet been examined in BD. Addressing this gap is critical for determining whether social support can function as a protective resource that mitigates the adverse impact of CM on NSSI in BD. Prior research has seldom examined how cognitive vulnerabilities and protective factors jointly shape the pathway from CM to NSSI in BD.

**Figure 1:**
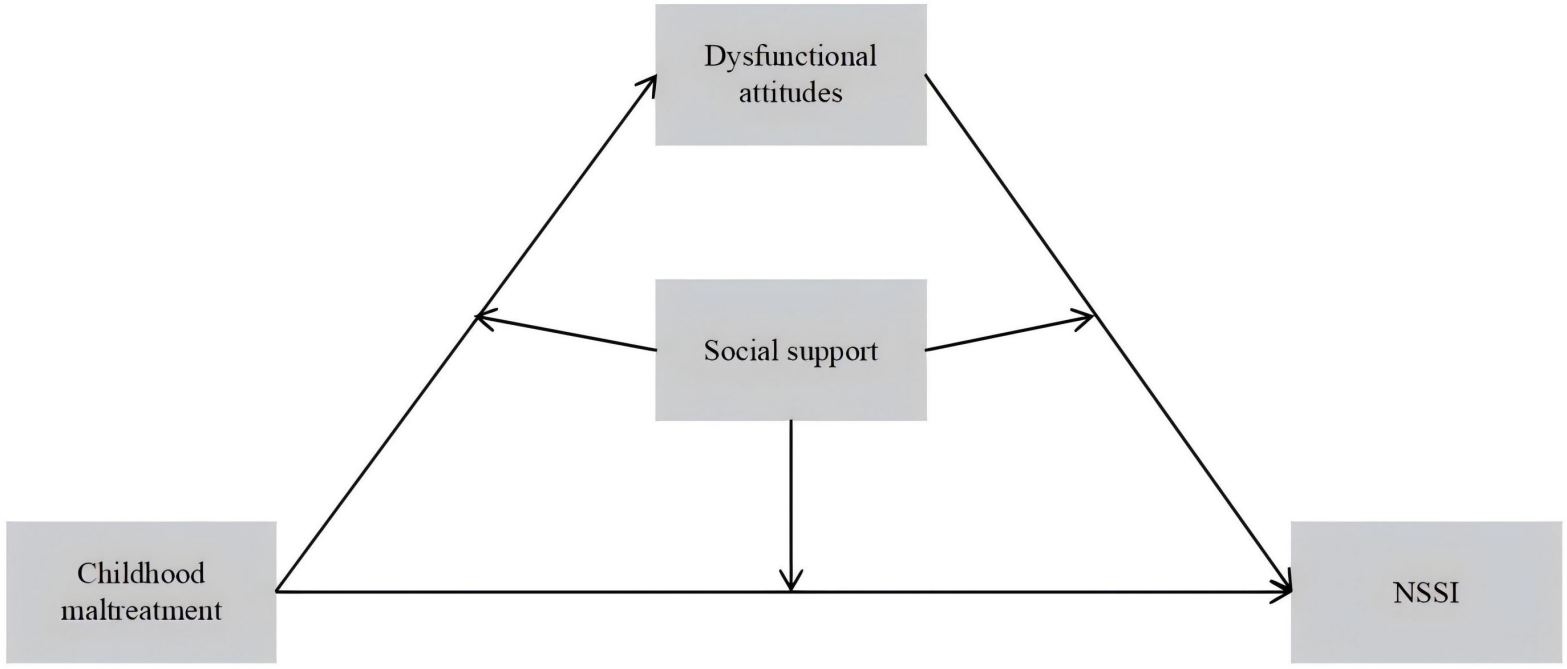
Proposed conceptual model.

To fill this gap, we developed a conceptual framework (**Figure 1**) that incorporates both a mediating mechanism and a moderating mechanism. Within this framework, we examined whether dysfunctional attitudes mediate the association between CM and NSSI in adolescents with BD, and whether social support moderates three key associations: CM with dysfunctional attitudes, CM with NSSI, and dysfunctional attitudes with NSSI. This integrative perspective is expected to refine trauma–cognition–behavior models and provide an empirical basis for mechanism-based prevention and intervention strategies tailored to high-risk BD populations. Based on this framework, the following hypotheses were proposed:

H1: CM is directly associated with NSSI.

H2: dysfunctional attitudes mediate the association between CM and NSSI.

H3: social support moderates the associations between (a) CM and NSSI, (b) CM and dysfunctional attitudes, and (c) dysfunctional attitudes and NSSI.

## 2 Methods

### 2.1 Participants

This cross-sectional study was conducted at Renmin Hospital of Wuhan University, Wuhan, Hubei Province, China, between March 28, 2024, and February 13, 2025. A total of 838 adolescents with a clinical diagnosis of BD were enrolled. Participants were recruited from both inpatient and outpatient psychiatric departments. Individuals with either BD type I or type II were eligible. Inclusion criteria were: (1) adolescents aged 14–19 years; (2) meeting the Diagnostic and Statistical Manual of Mental Disorders, Fifth Edition (DSM-5) criteria for BD (1); (3) confirmation of diagnosis by two board-certified psychiatrists using the Mini International Neuropsychiatric Interview (MINI) (24); (4) ability and willingness to provide informed consent. Exclusion criteria were: (1) comorbid severe psychiatric disorders (e.g., schizophrenia); (2) major neurological diseases; (3) severe physical illnesses; (4) pregnancy or lactation; and (5) inability to complete the assessment due to substantial cognitive or linguistic impairments.

All participants completed standardized self-report questionnaires and clinician-rated assessments administered by trained evaluators. The average evaluation time was approximately 20–30 minutes.

The study protocol was approved by the Ethics Committee of Renmin Hospital, Wuhan University (Approval No. WDRY24K039). All research procedures adhered to the principles outlined in the Declaration of Helsinki. Prior to data collection, written informed consent was obtained from all participants, who were clearly informed of their right to withdraw from the study at any time without negative consequences. Strict measures were implemented to ensure the confidentiality and protection of personal data.

### 2.2 Measures

#### 2.2.1 Compilation of general information

Basic demographic information was collected using a structured self-report questionnaire, including participants’ age, gender, and years of education. Clinical and psychological variables were assessed using standardized scales, as described in the following sections.

#### 2.2.2 Nonsuicidal self-injury

Nonsuicidal self-injury (NSSI) was assessed using the Ottawa Self-Injury Inventory (OSI), a prevalent self-report tool that records the incidence, frequency, techniques, psychological functions, and addictive characteristics of NSSI (25). Previous studies have demonstrated that the OSI shows adequate reliability and validity in university samples (26). This study defined NSSI status based on participants’ replies to the question, “In the past 6 months, how many times have you engaged in self-injury without suicidal intent?” Individuals who reported no instances of NSSI during this period (0 times) were classified as the non-NSSI group (coded as 0), whereas those who reported one or more instances (≥1 time) were designated as the NSSI group (coded as 1). This binary categorization strategy, predicated on the occurrence of NSSI during a defined period, corresponds with techniques utilized in prior empirical research (27, 28). The OSI demonstrated exceptional internal consistency in our sample, with a Cronbach’s α of 0.978, highlighting its appropriateness for evaluating NSSI among Chinese college students.

#### 2.2.3 Childhood maltreatment

The Childhood Trauma Questionnaire–Short Form (CTQ-SF), developed by Bernstein, is a retrospective self-report tool designed to assess adverse experiences before the age of 16 (29). It consists of 28 items rated on a 5-point Likert scale ranging from “never true” to “very often true”, with higher scores indicating greater severity of maltreatment (30). The instrument covers five domains of childhood adversity: emotional abuse, physical abuse, sexual abuse, emotional neglect, and physical neglect. The CTQ-SF has been extensively applied in both research and clinical contexts, and its psychometric properties have been well established in Chinese populations (31, 32). In the present study, the total scale demonstrated excellent internal consistency, with a Cronbach’s α of 0.863.

#### 2.2.4 Dysfunctional attitudes

The Chinese version of the Dysfunctional Attitudes Scale–Form A (DAS-A) was employed to assess maladaptive cognitive beliefs that may contribute to vulnerability to depression (13). The instrument consists of 40 self-report items, such as “I am nothing if a person I love does not love me,” and each item is rated on a 7-point Likert scale ranging from 1 (“totally disagree”) to 7 (“totally agree”). Total scores range from 40 to 280, with higher values indicating stronger endorsement of dysfunctional attitudes. Previous studies have demonstrated that the Chinese DAS-A possesses good reliability and validity across both clinical and non-clinical populations (33, 34). In the present sample, the internal consistency of the scale was satisfactory, with a Cronbach’s α of 0.935, supporting its suitability for use in this study.

#### 2.2.5 Social support

The Social Support Rating Scale (SSRS) is a multidimensional self-report instrument developed in China to assess perceived and received social support (35). This instrument includes 10 items that assess three key dimensions of social support: subjective support, objective support, and utilization of support. Items are rated on a 4-point Likert scale ranging from 1 (“none”) to 4 (“full support”), yielding a total score between 12 and 66, with higher scores representing a greater level of perceived social support. The SSRS has been extensively employed in diverse Chinese populations, both clinical and community-based, and prior studies have confirmed its satisfactory reliability and validity (36). In the present sample, the scale demonstrated acceptable internal consistency, with a Cronbach’s α of 0.765, supporting its applicability in this study.

#### 2.2.6 Depression severity

The 24-item Hamilton Depression Rating Scale (HAMD-24), developed by Hamilton in 1960 and later revised, was used to assess the severity of depressive symptoms (37). In the present study, we adopted the 5-point version, in which each item is rated from 0 (“absent”) to 4 (“severe”), allowing for more fine-grained distinctions in symptom severity across patients. The total score is obtained by summing all items, with higher scores reflecting more severe depressive symptoms. In line with previous research, the Chinese version of the 24-item Hamilton Depression Rating Scale (HAMD-24) has demonstrated satisfactory psychometric properties in clinical populations, with reported internal consistency ranging from Cronbach’s α = 0.81 to 0.86, supporting its validity and applicability for assessing depressive symptoms among Chinese patients (38, 39). Consistent with this evidence, the internal consistency of the scale in the current sample was acceptable, with a Cronbach’s α of 0.869, supporting its applicability for the present investigation.

### 2.3 Data analysis

All statistical analyses were performed using SPSS version 26.0 (IBM Corp., Armonk, NY, USA) and Mplus version 8.3 (Muthén & Muthén, Los Angeles, CA, USA). All tests were two-sided; p < 0.05 was considered statistically significant. Missing data (3.1%) were handled using multiple imputation under the missing at random (MAR) assumption, generating five imputed datasets. First, descriptive statistics were computed: categorical variables were summarized as frequencies and percentages, whereas continuous variables with skewed distributions were presented as medians and interquartile ranges (IQRs). Spearman’s rank correlation coefficients were calculated to examine the bivariate associations among study variables. Second, mediation and moderated mediation models were estimated in Mplus 8.3 using robust maximum likelihood (MLR). To test the moderating effects of social support, three separate models were established to reduce potential multicollinearity among interaction terms and to allow clearer interpretation of the distinct role of social support. For the binary outcome (NSSI), numerical integration via the Monte Carlo method was applied. As bootstrap confidence intervals are unavailable in this setting, the significance of indirect and interaction effects was evaluated using 95% confidence intervals from the Delta method; effects were deemed significant when the intervals excluded zero. All regression coefficients are reported as standardized estimates (β).

## 3 Results

### 3.1 Descriptive statistics and correlations

A total of 838 participants were included in the analysis. Among them, 68.1% were female (n = 571), and the median age was 16 years (IQR = 14–19). The median number of years of education was 10 (IQR = 8–12). The median depressive severity score was 20.5 (IQR = 13–28), indicating a moderate level of symptoms. Additionally, 68.5% of participants (n = 574) reported a history of NSSI. Among those who engaged in NSSI, the majority (58.5%) reported more than four episodes, and the most common method of self-injury was cutting (53.1%), followed by punching or hitting objects (13.4%) and stabbing or scratching (10.5%). For continuous study variables, the median (IQR) scores were 51 (40–62) for CM, 165 (144–190) for dysfunctional attitudes, and 27 (23–33) for social support. Detailed demographic and clinical characteristics are presented in **Table 1**.

**Table 1:** Demographic and clinical characteristics of participants (N = 838).

Characteristic	Median/frequency	IQR/percentage
Gender		
Female	571	68.1%
Male	267	31.9%
Age	16	14–19
Years of Education	10	8–12
Depressive severity	20.5	13-28
Childhood maltreatment	51	40–62
Dysfunctional attitudes	165	144–190
Social support	27	23–33
NSSI		
No	264	31.5%
Yes	574	68.5%
NSSI frequency		
1	75	13.07%
2	42	7.32%
3	33	5.75%
4	88	15.33%
>4	336	58.53%
NSSI method		
Cutting	305	53.1%
Stabbing/Scratching	60	10.5%
Punching/Hitting objects	77	13.4%
hair pulling/skin picking	50	8.7%
Other	82	14.3%

IQR, Interquartile Range, NSSI, Non-suicidal self-injury.

**Table 2** presents the computed Spearman rank-order correlations among education, gender, age, CM, depressive severity, social support, dysfunctional attitudes, and NSSI, as the data did not meet the assumption of normality. To assess potential multicollinearity, we conducted collinearity diagnostics; all variance inflation factors (VIFs) were below 5, indicating no problematic multicollinearity.

**Table 2:** Spearman correlations among study variables.

Variable	Education	Gender	Age	CM	DS	SS	DA	NSSI
Education	1							
Gender	0.099**	1						
Age	0.871**	0.111**	1					
CM	-0.151**	-0.138**	-0.108**	1				
DS	-0.159**	-0.215**	-0.161**	0.360**	1			
SS	0.231**	0.026	0.262**	-0.387**	-0.343**	1		
DA	-0.214**	-0.176**	-0.225**	0.335**	0.461**	-0.363**	1	
NSSI	-0.254**	-0.258**	-0.285**	0.323**	0.415**	-0.270**	0.392**	1

Spearman’s ρ correlation coefficients are reported. CM, Childhood maltreatment; DS, Depressive severity; SS, Social support; DA, Dysfunctional attitudes; NSSI, Non-suicidal self-injury. **p < 0.01.

### 3.2 Testing for the mediation effect

The mediation model tested the indirect association between CM and NSSI through dysfunctional attitudes. As shown in **Table 3**, CM was significantly associated with NSSI (β = 0.220, SE = 0.041, p < 0.001, 95% CI [0.139, 0.287]). The indirect effect of CM on NSSI via dysfunctional attitudes was significant (β = 0.040, SE = 0.011, p < 0.01, 95% CI [0.017, 0.061]), accounting for 18.18% of the total effect. The direct effect of CM on NSSI remained significant after including dysfunctional attitudes in the model (β = 0.180, SE = 0.042, p < 0.001, 95% CI [0.097, 0.263]). These results indicate that dysfunctional attitudes partially mediate the association between CM and NSSI.

**Table 3:** Analysis of the mediating effect of dysfunctional attitudes.

Effect	β	SE.	Est./SE	P-value	Bootstrap 95% CI (lower)	Bootstrap 95% CI (upper)	Proportion of total effect (%)
Total Effect	0.220	0.041	5.363	0.000	0.139	0.287	100
Direct Effect	0.180	0.042	4.258	0.000	0.097	0.263	81.82
Indirect Effect	0.040	0.011	3.467	0.001	0.017	0.061	18.18

### 3.3 Testing for the moderation effect

The results of the moderated mediation analyses are presented in **Table 4**. The three interaction terms were tested in separate regression models to avoid multicollinearity and interpretational ambiguity. In Model 1, social support was examined as a moderator of the relationship between CM and dysfunctional attitudes. The moderating effect of social support on the association between CM and dysfunctional attitudes was insignificant (β = 0.058, SE = 0.038, p > 0.05, 95% CI [–0.018, 0.121]). In Model 2, social support was examined as a moderator of the direct effect of CM on NSSI. The moderating effect of social support on the association between CM and NSSI was insignificant (β = –0.074, SE = 0.043, p > 0.05, 95% CI [–0.158, 0.011]). In Model 3, social support was examined as a moderator of the relationship between dysfunctional attitudes and NSSI. The moderating effect of social support on the association between dysfunctional attitudes and NSSI was statistically significant (β = –0.082, SE = 0.041, p < 0.05, 95% CI [–0.162, –0.015]). Higher levels of social support weakened the positive association between dysfunctional attitudes and NSSI.

**Table 4:** Moderating effects of social support: results from three regression models.

Model	Outcome variable	Predictor	Estimate	SE.	Est./SE	P-value	Bootstrap 95% CI (lower)	Bootstrap 95% CI (upper)
Model1 (SS moderates CM→DA)	DA	CM	0.164	0.037	4.403	0.000	0.091	0.237
		SS	-0.128	0.041	-3.149	0.002	-0.207	-0.048
		CM*SS	0.058	0.038	1.501	0.133	-0.018	0.121
	NSSI	CM	0.180	0.042	4.259	0.000	0.097	0.263
		DA	0.194	0.042	4.628	0.000	0.112	0.276
Model2 (SS moderates CM→NSSI)	DA	CM	0.202	0.035	5.706	0.000	0.133	0.272
	NSSI	CM	0.184	0.043	4.247	0.000	0.099	0.269
		DA	0.192	0.042	4.621	0.000	0.111	0.274
		SS	-0.056	0.042	-1.324	0.186	-0.164	0.027
		CM*SS	-0.074	0.043	-1.709	0.088	-0.158	0.011
Model3 (SS moderates DA→NSSI)	DA	CM	0.202	0.035	5.706	0.000	0.133	0.272
	NSSI	CM	0.175	0.044	4.021	0.000	0.090	0.261
		DA	0.201	0.043	4.711	0.000	0.117	0.284
		SS	-0.065	0.042	-1.542	0.123	-0.148	0.018
		DA*SS	-0.082	0.041	-2.016	0.044	-0.162	-0.015

SS, Social support; CM, Childhood maltreatment; DA, Dysfunctional attitudes; NSSI, Non-suicidal self-injury.

**Figure 2:**
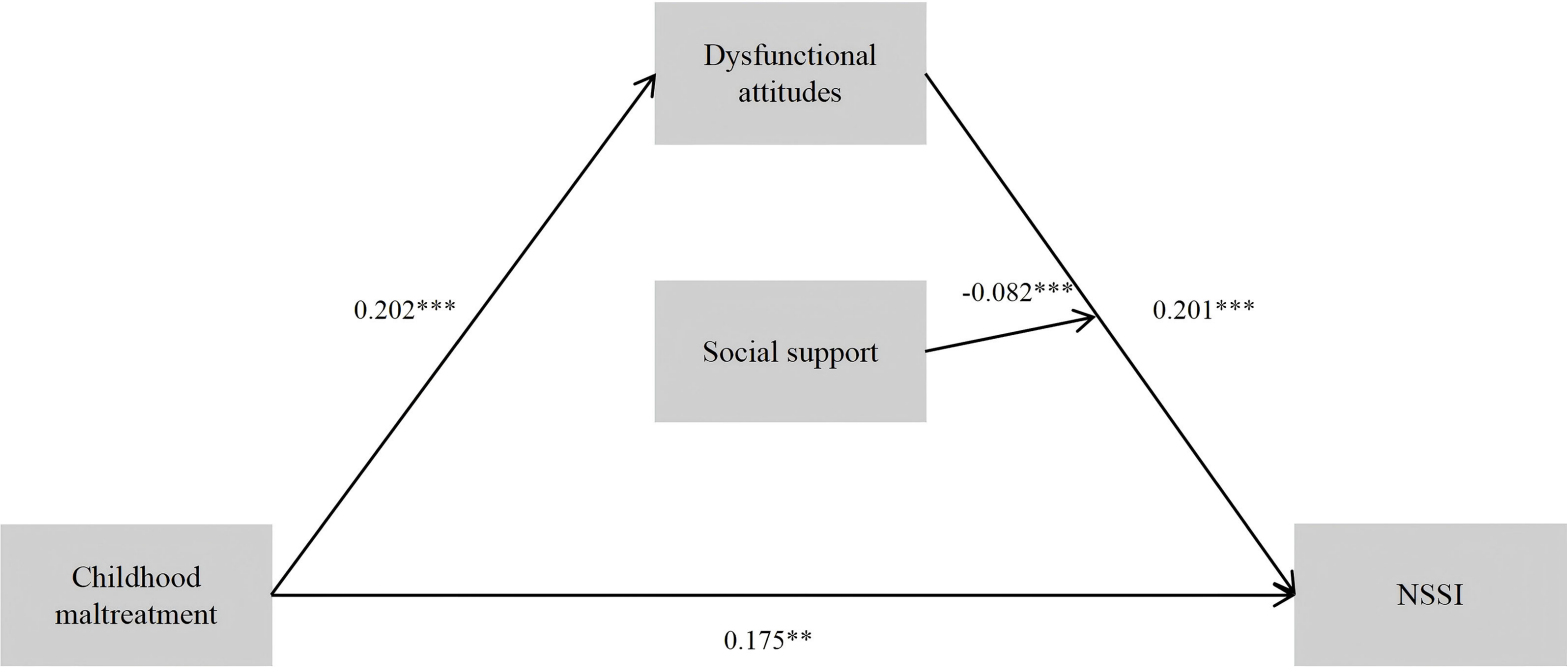
Standardized path diagram of the final moderated mediation model tested in Model 3.

**Figure 2** illustrates Model 3. CM was positively associated with dysfunctional attitudes, and both CM and dysfunctional attitudes significantly predicted NSSI. Social support significantly moderated the association between dysfunctional attitudes and NSSI, such that higher levels of social support weakened this positive relationship.

### 3.4 Simple slopes analyses

**Figure 3:**
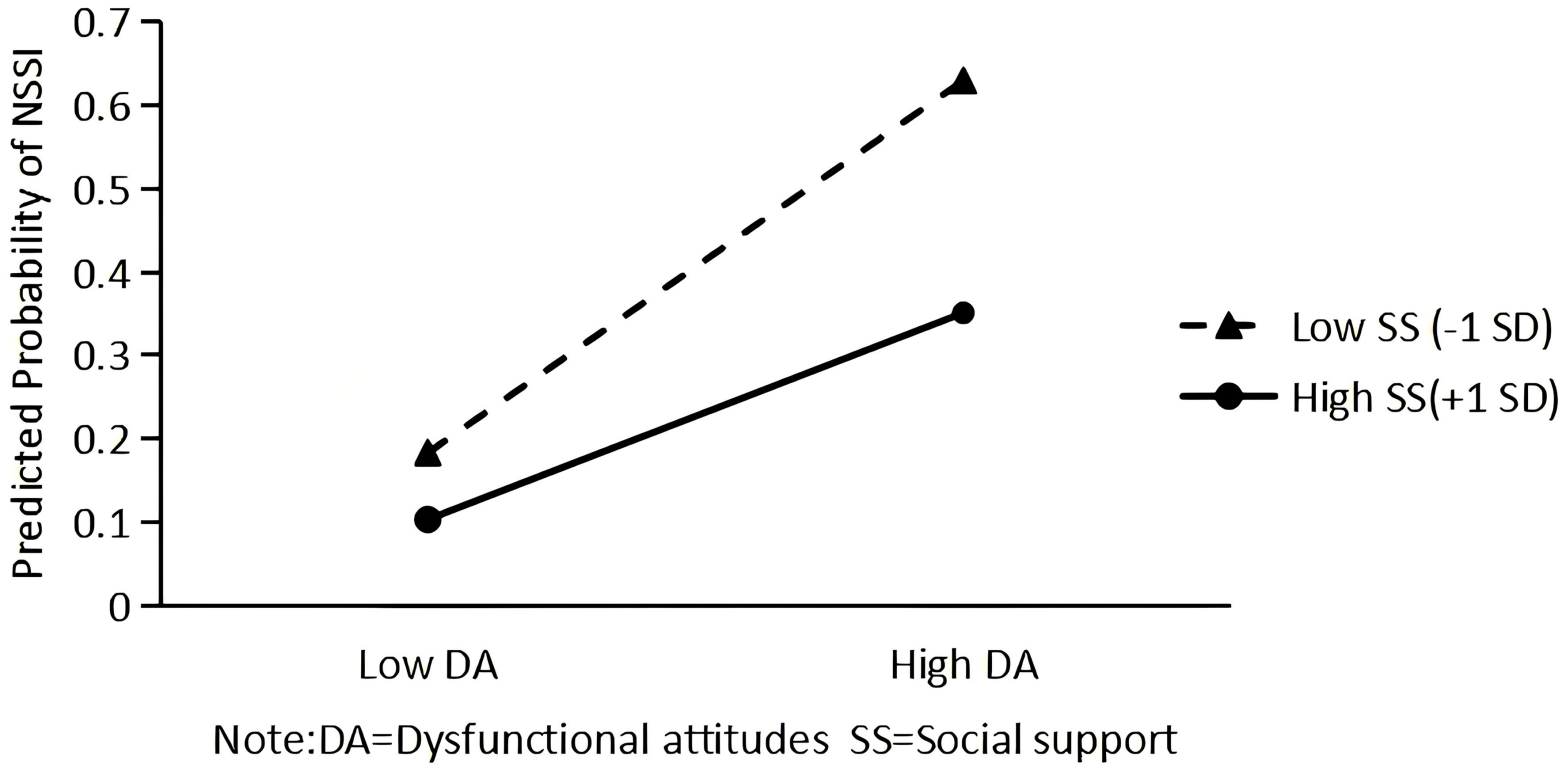
Simple slope plot illustrating the moderating effect of social support on the association between dysfunctional attitudes and NSSI.

To further probe the significant moderating effect of social support on the association between dysfunctional attitudes and NSSI (Model 3), a simple slopes analysis was conducted (see **Figure 3**). At low levels of social support (–1 SD), dysfunctional attitudes positively predicted NSSI with a steeper slope, indicating that individuals with lower social support were more vulnerable to the detrimental effects of dysfunctional attitudes. At high levels of social support (+1 SD), the effect of dysfunctional attitudes on non-suicidal self-injury was weaker, suggesting that higher social support buffered this risk pathway. The graphical pattern confirmed that social support mitigated the adverse influence of dysfunctional attitudes on the likelihood of engaging in NSSI.

## 4 Discussion

Using a clinically diagnosed sample of adolescents with BD, the present study investigated the mechanisms linking CM and NSSI by testing dysfunctional attitudes as a mediator and social support as a moderator. Three key contributions were identified. First, to our knowledge, this is among the first study to integrate CM, dysfunctional attitudes, social support, and NSSI into a unified moderated mediation framework and to validate this model in a BD population. Second, dysfunctional attitudes were shown to mediate the association between CM and NSSI, highlighting maladaptive cognition as a proximal mechanism through which early adversity translates into self-injurious behavior. Third, social support demonstrated a pathway-specific moderating effect, buffering only the link between dysfunctional attitudes and NSSI, but not the associations between CM and dysfunctional attitudes or between CM and NSSI. Together, these findings enrich the understanding of risk and protective processes underlying self-injury in BD and provide culturally relevant insights for clinical intervention in China, where mental health resources remain limited but traditional values emphasize family and interpersonal support as vital sources of resilience.

### 4.1 The direct effect of childhood maltreatment on NSSI

The present study demonstrated a direct association between CM and NSSI in adolescents with BD. Large-scale epidemiological and community-based studies of adolescents have demonstrated that experiences of childhood abuse or neglect significantly increase the risk of self-injurious behavior (40). Clinical investigations in patients with MDD have further confirmed that childhood trauma is associated with more frequent and severe NSSI (41). Within adolescent BD populations, numerous studies have similarly reported that CM exerts a robust direct effect on self-injury, even after controlling for affective instability, impulsivity, and comorbid symptoms (42). Other investigations suggest that CM may also act indirectly, through mechanisms such as emotion dysregulation, impulsivity, and maladaptive personality traits (43). Taken together, these findings indicate that the direct effect observed in the current study is both consistent with and extends previous research. Clinically, these results highlight the necessity of routine trauma screening and trauma-informed interventions in BD, particularly in China, where the assessment of childhood trauma is often overlooked in psychiatric practice.

### 4.2 The Mediating role of dysfunctional attitudes

The present study demonstrated that dysfunctional attitudes partially mediated the relationship between CM and NSSI in adolescents with BD, supporting H2. Previous studies have shown that experiences of CM foster negative beliefs about the self and others, reduce cognitive flexibility, and often lead to rigid, maladaptive cognitive patterns, particularly dysfunctional attitudes, with emotional abuse and neglect identified as key predictors (44, 45). Dysfunctional attitudes may adversely affect individuals’ behavioral regulation by reinforcing self-critical beliefs and hopeless interpretations, thereby lowering coping efficacy and increasing vulnerability to NSSI (46). CM may heighten the risk of NSSI by exacerbating dysfunctional attitudes, leading to rigid negative beliefs and maladaptive interpretative patterns that undermine positive self-appraisals and problem-solving abilities. This may, in turn, increase reliance on maladaptive coping strategies. The present study’s findings are highly consistent with the cognitive vulnerability model (47), which suggests that early adverse experiences contribute to the formation of rigid, maladaptive belief systems, thereby increasing susceptibility to psychopathology and the likelihood of maladaptive behaviors. In this study, CM functioned as a distal risk factor for NSSI, exerting its influence indirectly through the mediating mechanism of a proximal cognitive vulnerability—dysfunctional attitudes. As a concrete manifestation of cognitive vulnerability, dysfunctional attitudes intensify negative appraisals of the self and the environment, making individuals less likely to adopt adaptive coping strategies and more prone to maladaptive ones such as NSSI (16).

Importantly, the indirect effect accounted for approximately 18% of the total effect, representing a small-to-moderate mediation effect. This suggests that although dysfunctional attitudes play a meaningful role in linking CM to NSSI, additional psychological, social, and environmental factors likely contribute to the development of self-injurious behaviors in adolescents with BD.

The findings of the present study identified a novel mediating mechanism linking CM to NSSI through dysfunctional attitudes, providing new insights for developing interventions to reduce self-injury risk in adolescents with BD and a history of CM. Theoretically, these findings suggest that cognitive vulnerability frameworks are not only applicable to depressive disorders but also extend to explaining self-injurious behaviors in individuals with BD. Practically, the results highlight dysfunctional attitudes as a critical intervention target, with approaches such as cognitive-behavioral therapy and schema-focused therapy offering promising avenues for reducing self-injury risk among trauma-exposed BD patients. Notably, in the Chinese context, where the treatment of BD remains predominantly pharmacological and structured psychotherapeutic resources are limited, the present findings underscore the urgency of integrating cognitive restructuring strategies into routine clinical practice.

### 4.3 The moderating influence of social support

The present study found that perceived social support moderated only the association between dysfunctional attitudes and NSSI, but not the links from CM to dysfunctional attitudes or from CM to NSSI. This suggests that while social support may not buffer the direct effects of CM on maladaptive cognitions or NSSI, it becomes particularly influential when dysfunctional attitudes have already developed and begin to manifest in self-injurious behaviors. These findings partially supported hypothesis H3. This suggests that the buffering role of social support is not uniformly distributed across all hypothesized associations but operates specifically at the level of translating maladaptive cognitions into behavioral outcomes (48). The stability of such rigid and maladaptive belief systems is consistent with the present finding that social support did not moderate the effect of CM on dysfunctional attitudes. Previous research has shown that adverse childhood experiences (ACEs) and low social support significantly increase the risk of NSSI and suicidal behaviors among adolescents, suggesting that social support plays a critical protective role in buffering the adverse impact of early adversity on self-injurious outcomes (49). For example, among Chinese undergraduates, social support partially mediated the association between CM and NSSI, with the mediating effect being particularly pronounced among only-children, in whom social support reduced the impact of emotional and physical abuse on NSSI (50). However, very few studies have reported that social support moderates the direct effect of CM on NSSI. This is consistent with the present results, which showed that social support was not a significant moderator of the direct CM–NSSI association.

In contrast, social support significantly moderated the association between dysfunctional attitudes and NSSI. Specifically, the dysfunctional attitudes–NSSI link was stronger under low social support and markedly attenuated under high social support. This proves that supportive interpersonal resources can reduce how maladaptive cognitions are translated into self-injurious behaviors. Research has shown that dysfunctional attitudes, conceptualized as rigid and maladaptive beliefs, amplify negative self-appraisals, reduce cognitive flexibility, and increase vulnerability to maladaptive coping (51). Other forms of maladaptive cognitive styles, such as rumination and catastrophizing, have also been shown to heighten the impact of emotion dysregulation on NSSI, further supporting the role of cognitive vulnerability in self-injurious behaviors (52).

Meanwhile, higher levels of social support have been repeatedly linked to lower rates of self-injury and suicidality, underscoring its protective role in mitigating the adverse effects of psychological vulnerabilities (53). The present findings integrate these strands of evidence by showing that, among individuals with elevated dysfunctional attitudes, high social support significantly reduces the strength of the dysfunctional attitudes–NSSI association. This pattern is consistent with the stress-buffering model (18), which posits that adequate interpersonal resources can alleviate the psychological strain induced by cognitive vulnerabilities, reducing the likelihood of maladaptive cognitions being enacted as self-injurious behavior.

Together, these findings extend prior literature by identifying a boundary condition for the protective role of social support. While social support may not prevent the development of dysfunctional cognitions, it can substantially reduce their behavioral consequences. Practically, the results highlight the importance of integrating social support enhancement into clinical interventions, such as structured family programs, peer support, and therapy modules that strengthen interpersonal effectiveness. In the Chinese context, where treatment for BD remains predominantly pharmacological and psychotherapeutic resources are limited, enhancing social support may represent an accessible and culturally congruent strategy to reduce self-injury risk.

### 4.4 Limitations and future directions

First, the sample consisted exclusively of adolescents with clinically diagnosed bipolar disorder, and no differentiation was made between BD subtypes (e.g., BD-I and BD-II) or mood states at the time of assessment. This limits the specificity of the conclusions regarding heterogeneity within BD, and future studies should examine subtype- and mood state–related differences to provide more nuanced insights. Second, although the moderated mediation model elucidated the roles of CM, dysfunctional attitudes, and social support in NSSI, other potentially relevant psychological and environmental factors—such as emotion dysregulation, interpersonal difficulties, and impulsivity—were not included. Future research should incorporate these variables to provide a more comprehensive understanding of the mechanisms underlying self-injurious behaviors in BD. Third, the cross-sectional design and reliance on self-report measures limit the ability to draw causal inferences and may have introduced recall bias or shared method variance. Incorporating longitudinal designs, clinician-rated instruments, behavioral tasks, or multi-informant reports would improve measurement accuracy and clarify directionality. Finally, although this study was conducted in China, culturally specific mechanisms such as collectivism, family obligation, and stigma surrounding mental illness were not directly examined. Future studies should explore these sociocultural factors to understand better potential pathways unique to Chinese or other collectivist cultural contexts.

## 5 Conclusion

This study among adolescents with BD demonstrated that CM increases the risk of NSSI through dysfunctional attitudes, and that perceived social support moderates this association. Specifically, social support significantly attenuated the dysfunctional attitudes–NSSI link, but did not moderate the associations between CM and dysfunctional attitudes or NSSI. These findings identify dysfunctional attitudes as a key cognitive mechanism linking CM to NSSI in BD, and highlight social support as a conditional protective factor that operates primarily at the behavioral level. Clinically, the results underscore the importance of routinely assessing CM in BD patients, targeting dysfunctional attitudes through cognitive-behavioral and schema-focused interventions, and incorporating strategies to enhance social support into treatment planning. In the Chinese context, where BD management remains predominantly pharmacological and systematic psychotherapeutic resources are limited, integrating cognitive restructuring with support-based interventions may represent an accessible and culturally relevant approach to reducing the long-term risk of NSSI.

## Data Availability

The raw data supporting the conclusions of this article will be made available by the authors, without undue reservation.

## References

[1] Battle DE . Diagnostic and statistical manual of mental disorders (DSM). Codas. (2013) 25:191–2. doi: 10.1590/s2317-17822013000200017, PMID: 24413388

[2] Klonsky ED Victor SE Saffer BY . Nonsuicidal self-injury: what we know, and what we need to know. Can J Psychiatry. (2014) 59:565–8. doi: 10.1177/070674371405901101, PMID: 25565471 PMC4244874

[3] Yang SY Lee D Jeong H Cho Y Ahn JE Hong KS . Comparison of patterns of non-suicidal self-injury and emotion dysregulation across mood disorder subtypes. Front Psychiatry. (2022) 13:757933. doi: 10.3389/fpsyt.2022.757933, PMID: 35633812 PMC9133457

[4] Cui L Zhu Y Li Y Zhou J Xu G Pan M . A national survey of suicidality and non-suicidal self-injury in bipolar disorder: insights from network analysis. BMC Psychiatry. (2025) 25:297. doi: 10.1186/s12888-025-06750-2, PMID: 40155853 PMC11951841

[5] Zhong R Wang Z Zhu Y Wu X Wang X Wu H . Prevalence and correlates of non-suicidal self-injury among patients with bipolar disorder: A multicenter study across China. J Affect Disord. (2024) 367:333–41. doi: 10.1016/j.jad.2024.08.231, PMID: 39233245

[6] Esposito-Smythers C Goldstein T Birmaher B Goldstein B Hunt J Ryan N . Clinical and psychosocial correlates of non-suicidal self-injury within a sample of children and adolescents with bipolar disorder. J Affect Disord. (2010) 125:89–97. doi: 10.1016/j.jad.2009.12.029, PMID: 20089313 PMC2888943

[7] Lippard ETC Nemeroff CB . The devastating clinical consequences of child abuse and neglect: increased disease vulnerability and poor treatment response in mood disorders. AJP. (2020) 177:20–36. doi: 10.1176/appi.ajp.2019.19010020, PMID: 31537091 PMC6939135

[8] Zhang S Lin X Yang T Zhang S Pan Y Lu J . Prevalence of childhood trauma among adults with affective disorder using the Childhood Trauma Questionnaire: A meta-analysis. J Affect Disord. (2020) 276:546–54. doi: 10.1016/j.jad.2020.07.001, PMID: 32871685

[9] Yang L Du X Huang M . Childhood maltreatment and non-suicidal self-injury: the mediating role of mentalization and depression. Eur J Psychotraumatol. (2025) 16:2466279. doi: 10.1080/20008066.2025.2466279, PMID: 39995338 PMC11864010

[10] Calvo N Lugo-Marín J Oriol M Pérez-Galbarro C Restoy D Ramos-Quiroga J-A . Childhood maltreatment and non-suicidal self-injury in adolescent population: A systematic review and meta-analysis. Child Abuse Negl. (2024) 157:107048. doi: 10.1016/j.chiabu.2024.107048, PMID: 39332140

[11] Wang L Liu J Yang Y Zou H . Prevalence and risk factors for non-suicidal self-injury among patients with depression or bipolar disorder in China. BMC Psychiatry. (2021) 21:389. doi: 10.1186/s12888-021-03392-y, PMID: 34348675 PMC8335871

[12] Zhang Y Hu Z Hu M Lu Z Yu H Yuan X . Effects of childhood trauma on nonsuicidal self-injury in adolescent patients with bipolar II depression. Brain and Behavior (2022) 12:e2771. doi: 10.1002/brb3.2771, PMID: 36168882 PMC9660408

[13] Weissman AN Beck AT . Development and validation of the dysfunctional attitude scale: A preliminary investigation. Toronto, Canada (1978). Available online at: https://eric.ed.gov/?id=ED167619.

[14] Reilly LC Ciesla JA Felton JW Weitlauf AS Anderson NL . Cognitive vulnerability to depression: A comparison of the weakest link, keystone and additive models. Cogn Emotion. (2012) 26:521–33. doi: 10.1080/02699931.2011.595776, PMID: 21851251 PMC4083570

[15] Poletti S Colombo C Benedetti F . Adverse childhood experiences worsen cognitive distortion during adult bipolar depression. Compr Psychiatry. (2014) 55:1803–8. doi: 10.1016/j.comppsych.2014.07.013, PMID: 25194467

[16] Sun T Liu J Wang H Yang BX Liu Z Liu J . Risk prediction model for non-suicidal self-injury in Chinese adolescents with major depressive disorder based on machine learning. NDT. (2024) 20:1539–51. doi: 10.2147/NDT.S460021, PMID: 39139655 PMC11319100

[17] Dou W Yu X Fang H Lu D Cai L Zhu C . Family and psychosocial functioning in bipolar disorder: the mediating effects of social support, resilience and suicidal ideation. Front Psychol. (2022) 12:807546. doi: 10.3389/fpsyg.2021.807546, PMID: 35153929 PMC8832135

[18] Cohen S Wills TA . Stress, social support, and the buffering hypothesis. psychol Bull. (1985) 98:310–57. doi: 10.1037/0033-2909.98.2.310 3901065

[19] Davis A Kallivayalil R . Stressful life events and social support in Bipolar disorder Cross-sectional study from South India shows stressful life events and greater severity of stress in the pre-onset period as risk factors for relapse. GPA. (2023) 6:102–20. doi: 10.52095/gpa.2023.6594.1069

[20] Zhang M-Z Shi J-X Rao W-M Chen R Yang H-G Wu N-J . The mediating effects of social support on the association between depression and life satisfaction among patients with schizophrenia or bipolar disorder. Medicine. (2023) 102:e33531. doi: 10.1097/MD.0000000000033531, PMID: 37083814 PMC10118370

[21] Guo Y Peng S Liu Q Wang W Lu C Jiang X . Adverse childhood experiences and adolescent non-suicidal self-injury: The role of social support in a national survey on sexual orientation and gender expression. Child Abuse Negl. (2025) 167:107576. doi: 10.1016/j.chiabu.2025.107576, PMID: 40561637

[22] Christoffersen MN Møhl B DePanfilis D Vammen KS . Non-Suicidal Self-Injury—Does social support make a difference? An epidemiological investigation of a Danish national sample. Child Abuse Negl. (2015) 44:106–16. doi: 10.1016/j.chiabu.2014.10.023, PMID: 25435107

[23] Xu W Shen X McDonnell D Wang J . Childhood maltreatment and suicidal ideation among Chinese adolescents: Moderated mediation effect of perceived social support and maladaptive cognitive emotion regulation strategies. Child Abuse Negl. (2024) 151:106732. doi: 10.1016/j.chiabu.2024.106732, PMID: 38503245

[24] Amorim P Lecrubier Y Weiller E Hergueta T Sheehan D . DSM-IH-R Psychotic Disorders: procedural validity of the Mini International Neuropsychiatric Interview (MINI). Concordance and causes for discordance with the CIDI. Eur Psychiatry. (1998) 13:26–34. doi: 10.1016/S0924-9338(97)86748-X, PMID: 19698595

[25] Martin J Cloutier PF Levesque C Bureau J-F Lafontaine M-F Nixon MK . Psychometric properties of the functions and addictive features scales of the Ottawa Self-Injury Inventory: A preliminary investigation using a university sample. psychol Assess. (2013) 25:1013–8. doi: 10.1037/a0032575, PMID: 23647037

[26] Guérin-Marion C Martin J Deneault A-A Lafontaine M-F Bureau J-F . The functions and addictive features of non-suicidal self-injury: A confirmatory factor analysis of the Ottawa self-injury inventory in a university sample. Psychiatry Res. (2018) 264:316–21. doi: 10.1016/j.psychres.2018.04.019, PMID: 29665561

[27] Zhang F Cloutier PF Yang H Liu W Cheng W Xiao Z . Non-suicidal self-injury in Shanghai inner bound middle school students. Gen Psych. (2019) 32:e100083. doi: 10.1136/gpsych-2019-100083, PMID: 31552387 PMC6738666

[28] Glenn CR Klonsky ED . Prospective prediction of nonsuicidal self-injury: A 1-year longitudinal study in young adults. Behav Ther. (2011) 42:751–62. doi: 10.1016/j.beth.2011.04.005, PMID: 22036002 PMC4433036

[29] Bernstein DP Fink L Handelsman L Foote J Lovejoy M Wenzel K . Initial reliability and validity of a new retrospective measure of child abuse and neglect. Am J Psychiatry. (1994) 151:1132–6. doi: 10.1176/ajp.151.8.1132, PMID: 8037246

[30] Bernstein DP Stein JA Newcomb MD Walker E Pogge D Ahluvalia T . Development and validation of a brief screening version of the Childhood Trauma Questionnaire. Child Abuse Negl. (2003) 27:169–90. doi: 10.1016/s0145-2134(02)00541-0, PMID: 12615092

[31] Cruz D . Childhood trauma questionnaire-short form: evaluation of factor structure and measurement invariance. J Child Adolesc Trauma. (2023) 16:1099–108. doi: 10.1007/s40653-023-00556-8, PMID: 38045834 PMC10689687

[32] Jiang W-J Zhong B-L Liu L-Z Zhou Y-J Hu X-H Li Y . Reliability and validity of the Chinese version of the Childhood Trauma Questionnaire-Short Form for inpatients with schizophrenia. PloS One. (2018) 13:e0208779. doi: 10.1371/journal.pone.0208779, PMID: 30543649 PMC6292582

[33] Yu T-F Liu L Shang L-N Xu F-F Chen Z-M Qian L-J . Dysfunctional attitudes, social support, negative life events, and depressive symptoms in Chinese adolescents: A moderated mediation model. World J Psychiatry. (2024) 14:1671–80. doi: 10.5498/wjp.v14.i11.1671, PMID: 39564176 PMC11572672

[34] Wong DFK Chan KS Lau Y . The reliability and validity of the Chinese version of the dysfunctional attitudes scale form a (Das-A) in a community sample. Int J Psychiatry Med. (2008) 38:141–52. doi: 10.2190/PM.38.2.b, PMID: 18724566

[35] Xiao SY . Theoretical foundation and research application of the Social Support Rating Scale. Chin J Ment Health. (1994) 8:98–100.

[36] Yuan L Zhao Z . Resilience, self-efficacy, social support, and quality of life in patients with skin defects of the lower extremity after flap transplantation. Ann Palliat Med. (2021) 10:443–53. doi: 10.21037/apm-20-2432, PMID: 33545776

[37] Hamilton M . A rating scale for depression. J Neurol Neurosurg Psychiatry. (1960) 23:56–62. doi: 10.1136/jnnp.23.1.56, PMID: 14399272 PMC495331

[38] Wu C-Y Huang H-C Wu S-I Sun F-J Huang C-R Liu S-I . Validation of the Chinese SAD PERSONS Scale to predict repeated self-harm in emergency attendees in Taiwan. BMC Psychiatry. (2014) 14:44. doi: 10.1186/1471-244X-14-44, PMID: 24533537 PMC3942520

[39] Liu J Duan W Xiao Z Wu Y . The effectiveness of online group mindfulness-based cognitive therapy for outpatients with depression in China. J Affect Disord. (2024) 351:387–91. doi: 10.1016/j.jad.2024.01.223, PMID: 38281594

[40] Edinger A Fischer-Waldschmidt G Parzer P Brunner R Resch F Kaess M . The impact of adverse childhood experiences on therapy outcome in adolescents engaging in nonsuicidal self-injury. Front Psychiatry. (2020) 11:505661. doi: 10.3389/fpsyt.2020.505661, PMID: 33329074 PMC7672012

[41] Xie X Liu J Gong X Sun T Li Y Liu Z . Relationship between childhood trauma and nonsuicidal self-injury among adolescents with depressive disorder: mediated by negative life events and coping style. Neuropsychiatr Dis Treat. (2023) 19:2271–81. doi: 10.2147/NDT.S431647, PMID: 37905171 PMC10613422

[42] Marwaha S Briley PM Perry A Rankin P DiFlorio A Craddock N . Explaining why childhood abuse is a risk factor for poorer clinical course in bipolar disorder: a path analysis of 923 people with bipolar I disorder. Psychol Med. (2020) 50:2346–54. doi: 10.1017/S0033291719002411, PMID: 31530330 PMC7610181

[43] Li X Liu X-L Wang Y-J Zhou D-S Yuan T-F . The effects of childhood maltreatment on adolescent non-suicidal self-injury behavior: mediating role of impulsivity. Front Psychiatry. (2023) 14:1139705. doi: 10.3389/fpsyt.2023.1139705, PMID: 37304425 PMC10250706

[44] Jugessur R Zhang Y Qin X Wang M Lu X Sun J . Childhood maltreatment predicts specific types of dysfunctional attitudes in participants with and without depression. Front Psychiatry. (2021) 12:728280. doi: 10.3389/fpsyt.2021.728280, PMID: 34744822 PMC8568793

[45] Zhou E Ma S Kang L Zhang N Wang P Wang W . Psychosocial factors associated with anxious depression. J Affect Disord. (2023) 322:39–45. doi: 10.1016/j.jad.2022.11.028, PMID: 36375541

[46] Palmer-Cooper EC Woods C Richardson T . The relationship between dysfunctional attitudes, maladaptive perfectionism, metacognition and symptoms of mania and depression in bipolar disorder: The role of self-compassion as a mediating factor. J Affect Disord. (2023) 341:265–74. doi: 10.1016/j.jad.2023.08.117, PMID: 37633530

[47] Abramson LY Alloy LB Hankin BL Haeffel GJ MacCoon DG Gibb BE . “Cognitive vulnerability-stress models of depression in a self-regulatory and psychobiological context. In: Handbook of depression. The Guilford Press, New York, NY, US (2002). p. 268–94. doi: 10.1097/00005053-200301000-00022

[48] May T Younan R Pilkington PD . Adolescent maladaptive schemas and childhood abuse and neglect: A systematic review and meta-analysis. Clin Psychol Psychother. (2022) 29:1159–71. doi: 10.1002/cpp.2712, PMID: 35060262 PMC9544896

[49] Wan Y Chen R Ma S McFeeters D Sun Y Hao J . Associations of adverse childhood experiences and social support with self-injurious behaviour and suicidality in adolescents. Br J Psychiatry. (2019) 214:146–52. doi: 10.1192/bjp.2018.263, PMID: 30477603 PMC6429251

[50] Xu H Song X Wang S Zhang S Xu S Wan Y . Mediating effect of social support in the relationship between childhood abuse and non-suicidal self-injury among Chinese undergraduates: the role of only-child status. Int J Environ Res Public Health. (2019) 16:4023. doi: 10.3390/ijerph16204023, PMID: 31640165 PMC6843968

[51] Conway CC Slavich GM Hammen C . Dysfunctional attitudes and affective responses to daily stressors: separating cognitive, genetic, and clinical influences on stress reactivity. Cognit Ther Res. (2015) 39:366–77. doi: 10.1007/s10608-014-9657-1, PMID: 27041782 PMC4817852

[52] Ge Y Xiao Y Li M Yang L Song P Li X . Maladaptive cognitive regulation moderates the mediating role of emotion dysregulation on the association between psychosocial factors and non-suicidal self-injury in depression. Front Psychiatry. (2023) 14:1279108. doi: 10.3389/fpsyt.2023.1279108, PMID: 38098637 PMC10719840

[53] Darvishi N Poorolajal J Azmi-Naei B Farhadi M . The role of social support in preventing suicidal ideations and behaviors: A systematic review and meta-analysis. J Res Health Sci. (2024) 24:e00609. doi: 10.34172/jrhs.2024.144, PMID: 39072545 PMC11264453

